# Neural Basis of Anticipatory Anxiety Reappraisals

**DOI:** 10.1371/journal.pone.0102836

**Published:** 2014-07-21

**Authors:** Shinpei Yoshimura, Yasumasa Okamoto, Atsuo Yoshino, Makoto Kobayakawa, Akihiko Machino, Shigeto Yamawaki

**Affiliations:** 1 Otemon Gakuin University, Ibaraki, Osaka, Japan; 2 Department of Psychiatry and Neurosciences, Division of Frontier Graduate School of Biomedical Sciences, Hiroshima University, Minami-ku, Hiroshima, Japan; University of Wuerzburg, Germany

## Abstract

Reappraisal is a well-known emotion regulation strategy. Recent neuroimaging studies suggest that reappraisal recruits both medial and lateral prefrontal brain regions. However, few studies have investigated neural representation of reappraisals associated with anticipatory anxiety, and the specific nature of the brain activity underlying this process remains unclear. We used functional magnetic resonance imaging (fMRI) to investigate neural activity associated with reappraisals of transient anticipatory anxiety. Although transient anxiety activated mainly subcortical regions, reappraisals targeting the anxiety were associated with increased activity in the medial and lateral prefrontal regions (including the orbitofrontal and anterior cingulate cortices). Reappraisal decreased fear circuit activity (including the amygdala and thalamus). Correlational analysis demonstrated that reductions in subjective anxiety associated with reappraisal were correlated with orbitofrontal and anterior cingulate cortex activation. Reappraisal recruits medial and lateral prefrontal regions; particularly the orbitofrontal and anterior cingulate cortices are associated with successful use of this emotion regulation strategy.

## Introduction

Anticipatory anxiety is a psychological and physiological state associated with the anticipation of threatening stimuli [1], [2] Anticipatory anxiety can be viewed as an adaptive function that mobilizes the individual to cope with danger, such that people evaluate the potential danger involved in upcoming events and then take various forms of mental or physical action in order to mitigate risk or reduce unpleasant affect associated with the event. Anticipatory anxiety arises through complex cognitive-emotional processing. However, failure to adequately regulate such anxiety may result in an anxiety disorder. For example, patients with panic disorder feel excessive anxiety that is triggered when they anticipate having uncued panic attacks or panic attack in specific situations. This anticipatory anxiety might lead to avoidance behavior, thereby maintaining the patient's anxiety about future panic attacks and the perceived inability to cope with them.

On the other hand, people can regulate anxiety to reduce maladaptive avoidance behavior and protect themselves from being overwhelmed by excessive anxiety. Adequate control of anxiety or other negative emotions is an important psychological factor for mental health. Gross [3], [4] formulated a theory of emotion regulation, proposing various forms of emotion regulation strategy. One such strategy involves a form of cognitive regulation called “reappraisal.” This cognitive change strategy involves changing the meaning of a stimulus in a way that alters its emotional impact [5]. Reappraisal typically includes reconsideration or reframing of an emotional event in less emotional terms. This form of emotion regulation is emphasized in cognitive behavioral therapy [6].

Some research has reported that successful reappraisal reduces not just subjective affect but also associated physiological response [7], [8]. In addition, recent neuroimaging studies have revealed that prefrontal regions, including the medial, orbital, and lateral prefrontal cortices as well as the anterior cingulate region, are involved in the regulation of negative emotion [9], [10]. These prefrontal regions have functional connectivity to the amygdala [11]. Reappraisal may therefore be associated with top-down processing that is embodied by increased activity of the prefrontal region activity along with decreased activation of the amygdala.

Two studies have investigated brain functioning associated with cognitive regulation of anticipatory anxiety [8], [12]. Kalisch and colleagues [8] studied anticipatory anxiety associated with pain. Participants were instructed to manage their anxiety by thinking about a safe and relaxing place of their own choosing. Participants showed decreased medial prefrontal cortex activity during reappraisal of anticipatory anxiety over the course of long trial durations (15.6 s). The researchers argued that this brain activity change likely reflected detachment from self-consciousness. However, anxiety-related amygdala activation was not found in this study. Given previous findings that the amygdala plays an important role in anxiety processing, we believe that observing functional changes in the limbic system during reappraisal might be necessary in order to construct an accurate neural model of anticipatory anxiety reappraisal.

On the other hand, the study of Herwig et al. [12] examined the effects of a cognitive strategy on brain activity during anticipation of unpleasant visual stimuli. Participants performed reality checking during expectation of unpleasant stimuli. Herwig et al [12] found increased medial and dorsolateral prefrontal cortex activity during attempts at cognitive control of anticipatory anxiety. At the same time, cognitive control suppressed amygdala activity. These findings are consistent with models of cognitive control that posit that reappraisal techniques engage prefrontal circuitry, which in turn works to down-regulate limbic activity [10], [13]. Herwig et al. [12]did not collect emotion ratings from participants during anticipation of the negative stimuli; therefore the relationship between activation changes in the prefrontal and amygdala regions and emotional change as a function of reappraisal remains ambiguous. We added this subjective evaluation dimension to our experimental paradigm and examined the relationship between brain activity changes during reappraisal and reduction of subjective anxiety.

We used the intraepidermal stimulation method to elicit anticipatory anxiety [14]. On the basis of existing neural models of reappraisal, we hypothesized that reappraisal of anxiety would elicit increased prefrontal region activity, including activation of the medial prefrontal and dorsolateral cortices. In addition, reappraisal should provoke a corresponding decrease in amygdala activation.

## Materials and Methods

### Participants

Fifteen healthy students of Hiroshima University participated (mean age  = 23.3, SD = 2.2; 6 males, 9 females). They had normal vision and motor functioning, and none had any psychiatric disorders as screened using the Structural Clinical Interview for DSM disorders (SCID). All participants gave their written informed consent. The ethics committee of Hiroshima University approved the study protocol.

Procedure.

### Experimental design

**Figure 1:**
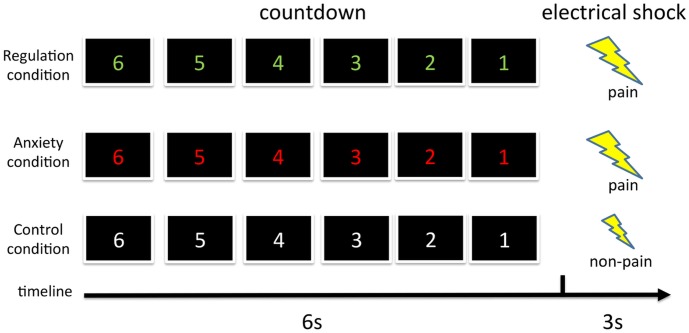
Schematic representation of experimental design.

The task included three conditions (Figure 1). In the regulation condition, participants received brief instruction describing the condition and then viewed a countdown displaying numerals from 6 down to 1 (presented in green). Following each countdown, participants received either a moderate pain stimulus, with a probability of 30% or a faint stimulus that elicited no perceived pain, with a probability of 70%. Participants were told these probabilities. Participants were instructed to regulate their anxiety by reinterpreting this anxious situation by such means as thinking realistically (e.g., Thinking about actual probability of receiving a pain stimulus). These reappraisal instructions were based on previous research using a reappraisal-based emotion regulation strategy [12]. In addition, participants were instructed that they should not try to distract themselves from the pain or the anxious situation. Before the fMRI scan, participants reported their reappraisal that they had used, and experimenter checked whether they had understood the reappraisal instructions. The anxiety condition was identical to the regulation condition, except that the countdown appeared in red, and participants were instructed to experience emotion as they naturally would, with no explicit attempts at regulation. In the control condition, participants viewed a white countdown and received only the faint stimulus.

Each condition consisted of four blocks, with one block composed of four trials. One trial included one countdown and one electrical stimulation, with a 12 sec Inter trial interval. Blocks were presented in a pseudorandom order. Following each block, participants evaluated their subjective anxiety during the countdown on a 10-point scale (1: *not at all anxious* - 10: *most anxious*). The total duration of the experiment was about 12 minutes. Visual stimuli were displayed on an MR-compatible back projection screen (Silent Vision SV-6011; Avotec, USA).

### Pain stimulation

We used the intraepidermal stimulation method [14] to induce minor pain at the superficial skin level. The original method was slightly modified to provide higher selectivity for nociceptor activation [15]. We used a stainless steel concentric bipolar needle electrode (Nihon Kohden, Tokyo, Japan), which permitted the selective stimulation of cutaneous A-delta fibers. The electrical stimuli used were 50 Hz constant double pulses of a 0.5 ms duration. The electrical stimuli were intended to evoke the feeling of receiving an injection. The needle electrode was exchanged for each participant. The constant current stimulator (SEN-8203; Nihon Kohden, Tokyo, Japan) was located outside the MRI room, and the electrode was connected to the stimulator via a magnet-compatible extension cable. Current intensity was determined before recordings were made. We stimulated the left forearm of each participant. We established stimulus current intensities for moderate pain (1 mA–5 mA) and faint stimulation (0.3 mA). Intensity of pain stimulation was adjusted for each participant's permissible range in order to elicit anticipatory anxiety. The insertion of the needle electrode caused no bleeding or visible damage to the skin of any participant.

### fMRI acquisition

The fMRI procedure was performed using a Symphony 1.5 tesla (Siemens, Munich, Germany). A total of 280 scans were performed for each participant using T2*-weighted, gradient echo, echo planar imaging (EPI) sequences. Each volume consisted of 34 slices, with a slice thickness of 4 mm with no gap, and covered the entire cerebral and cerebellar cortices. The time interval between two successive acquisitions of the same image (TR) was 3000 ms, the echo time (TE) was 46 ms, and the flip angle was 90°. The field of view (FOV) was 256 mm and the matrix size was 64×64, giving voxel dimensions of 4×4×4 mm. Scan acquisition was synchronized to the onset of each trial. After functional scanning, structural scans were acquired using a T1 - weighted gradient echo pulse sequence (TR = 12 ms; TE = 3.93 ms; flip angle 25°; FOV 256 mm; voxel dimensions of 1×1×1 mm), which facilitated localization.

### Data analysis

Image processing and statistical analyses were carried out using Statistical Parametric Mapping (SPM8). The first two and last single volumes were discarded because the MR signals were unsteady. All EPI images were spatially normalized using the Montreal Neurological Institute (MNI) T1 template for group analysis. Imaging data were corrected for motion and smoothed with an 8 mm full-width half-maximum Gaussian filter.

To perform image data analysis, a whole-brain voxel-by-voxel multiple linear regression model was employed at the individual participant level. The individual model comprised the covariate of no interest (realignment parameters). A general linear model analysis was then used to create contrast images for each participant, summarizing differences between conditions. We examined the following four contrasts to reveal activity associated with anticipatory anxiety and regulation: anxiety condition vs. control condition, regulation condition vs. control condition, anxiety condition vs. regulation condition, regulation condition vs. anxiety condition.

Using a group analysis based on a random effects model, we identified regions that exhibited significant responses using one-sample t-tests (uncorrected *p*<0.001, 20 voxels over).

In addition, to examine the possible relationship between reduction of anticipatory anxiety via reappraisal and brain activity in the regulation condition, we conducted a correlational analysis examining the relationship between anxiety reduction (subtracting anxiety ratings in the regulation condition from corresponding ratings in the anxiety condition with values averaged across the four blocks in each condition) and the first eigenvariate cluster for the regulation vs. anxiety condition comparison.

## Results

### Behavioral data (Figure 2)

**Figure 2:**
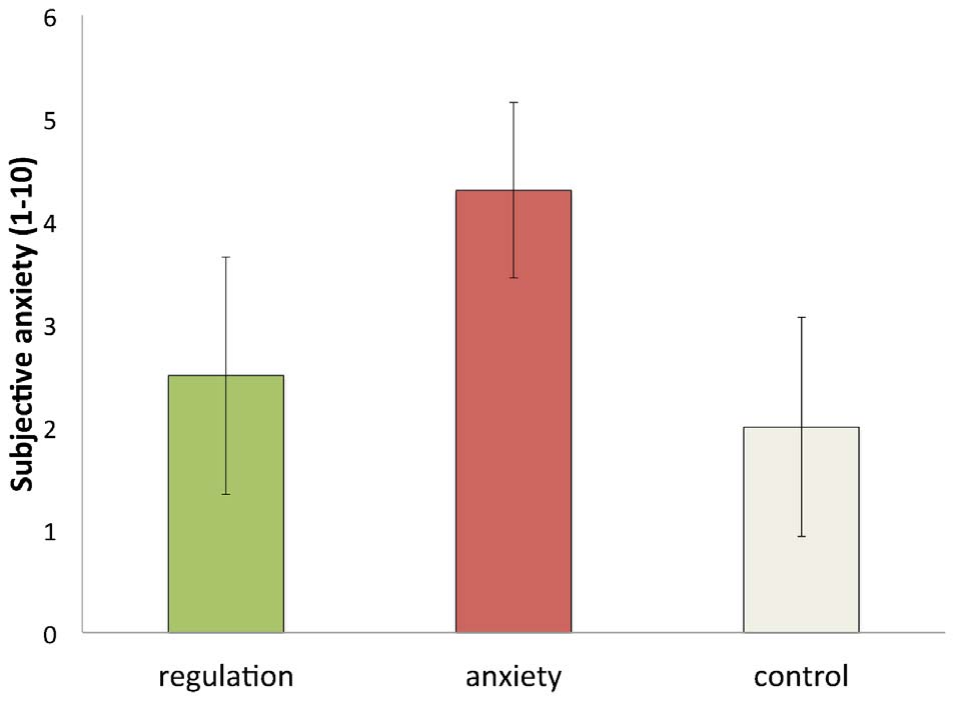
Subjective anxiety ratings from participants in each condition.

Repeated measures analysis of variance showed that participants indicated more subjective anxiety in the anxiety condition than in the other two conditions (all *p*s<0.01). There was no significant difference between subjective anxiety rating in the regulation condition and in the control condition. These subjective reports provide some validation of the experimental manipulation.

### fMRI data (Figure 3)

**Figure 3:**
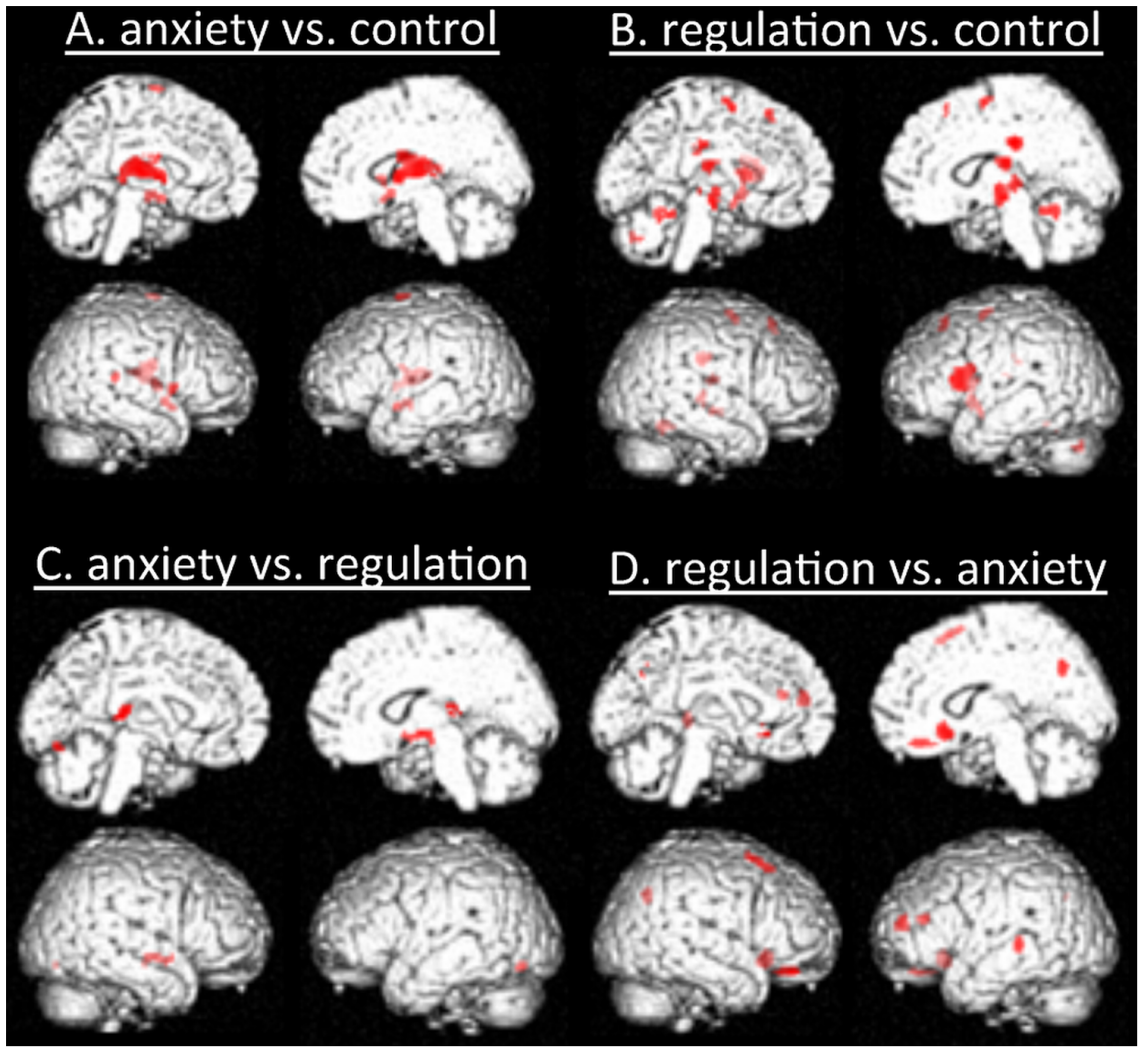
Statistical parametric maps showing activations for each contrast.

One-sample t-test results for each contrast are summarized in Table 1. The anxiety vs. control contrast exhibited activation of the right thalamus, right superior temporal cortex, right insula, left superior frontal cortex, right putamen, and left amygdala/hippocampus. On the other hand, the regulation vs. control contrast showed increased activity of the right middle cingulate cortex, left inferior frontal cortex, right thalamus, right cerebellum, left caudate, left supplemental motor cortex, and right superior medial frontal cortex.

**Table 1:** Anxiety or regulation-related activation.

condition	region	cluster extent	side	z-score	x	y	z
anxiety vs. control	Thalamus	846	R	4.85	4	−16	8
	Superior temporal cortex	23	R	4.24	52	−34	10
	Insula	54	R	4.07	42	10	2
	Superior frontal cortex	23	L	3.68	−12	−4	72
	Putamen	48	R	3.65	24	4	−8
	Amygdala/Hippocampus	74	L	3.64	−30	0	−12
regulation vs. control	Middle cingulate cortex	135	R	5.10	12	−28	30
	Inferior frontal cortex	181	L	4.57	−56	14	10
	Thalamus	124	R	4.31	4	−16	−10
	Thalamus	89	R	4.06	6	−18	18
	Cerebellum	82	R	3.86	8	−54	−18
	Hippocampus	54	R	3.82	14	−26	2
	Caudate	108	L	3.68	−14	8	12
	Supplemental motor cortex	45	L	3.53	0	−2	60
	Cerebellum	25	L	3.52	−16	−70	−36
	Superior medial frontal cortex	28	R	3.42	8	24	60
anxiety vs. regulation	Thalamus	56	L	4.18	0	−28	12
	Amygdala/Hippocampus	69	R	3.70	18	−12	−8
	Cerebellum	21	L	3.55	−18	−74	−16
regulation vs. anxiety	Middle orbitofrontal cortex	68	R	4.26	20	38	−18
	Anterior cingulate cortex	96	R	4.22	10	20	−10
	Middle frontal cortex	49	L	4.18	−26	50	16
	Middle frontal cortex	53	R	3.84	34	18	58
	Precuneus	46	R	3.67	12	−64	32
	Middle frontal cortex	30	L	3.62	−28	34	18
	Middle temporal cortex	40	L	3.54	−58	−34	0

Next, we conducted a direct comparison of the regulation and anxiety conditions to examine the effect of reappraisal on anticipatory anxiety. The anxiety vs. regulation contrast revealed right thalamus, right amygdala/hippocampus, and left cerebellum activation. The regulation vs. anxiety contrast exhibited activation of the right middle orbitofrontal cortex, right anterior cingulate cortex, left middle frontal cortex, and left middle temporal cortex.

**Figure 4:**
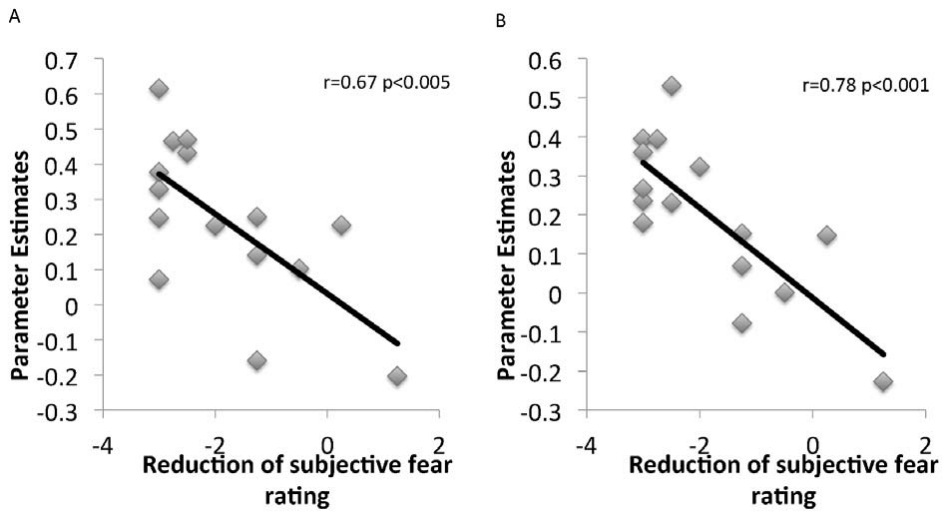
Scatter plot A shows the relationship between anxiety reduction as a result of reappraisal and activity of the anterior cingulate cortex. Scatterplot B shows the relationship between anxiety reduction as a result of reappraisal and activity of the orbitofrontal cortex.

Correlational analysis revealed that anxiety reduction as a result of reappraisal was significantly correlated with activation of the right middle orbitofrontal cortex (*r* = −0.78, *p*<0.001) and right anterior cingulate cortex (*r* = −0.67, *p*<0.005) in the regulation vs. anxiety contrast (Figure 4).

## Discussion

The aim of present study was to investigate neural correlates during reappraisal of transient anticipatory anxiety induced by administration of electrical pain stimuli. We found significant activation of limbic regions (including the amygdala and thalamus) in the anxiety condition as compared to the regulation and control conditions. In addition, reappraisal induced activity in the prefrontal and anterior cingulate cortices compared to the anxiety and control conditions. Anterior cingulate and orbitofrontal cortex activations during the regulation condition were negatively correlated with anxiety reduction by reappraisal.

Our results also revealed increased thalamus, insula, and amygdala activity in the anxiety vs. control contrast. These regions have been implicated in prior studies of anticipatory emotion, including anticipatory anxiety, which arises when one expercts a noxious stimulus [16]–[21]. Many studies have highlighted the amygdala as a key region involved in the so-called fear circuit. The amygdala may be involved in the detection of noxious stimuli during expectation-related processing, including expected pain [14]. Thalamus activation may play a role in threat expectancies as well. Herwig and colleagues [16] reported increased thalamus activation during the expectation of an emotional stimulus. Finally the insula has a wide range of reciprocal connections to prefrontal areas, the anterior cingulate cortex, thalamus, and amygdala [22], [23], and is thought to involve evaluation of somatosensory states that accompany emotional responses[18], [24], [25]. The increased insula activity observed in the present study is likely a consequence of the somatic sensations that would be expected to accompany anticipatory anxiety. Taken together, these results suggest that our use of relatively short trial durations successfully evoked brain activity associated with transient anticipatory anxiety (i.e., limbic activation).

Our results for the regulation vs. control contrast revealed increasing frontal region activity, including activation of the medial prefrontal cortex and inferior frontal cortex. This pattern of activation is consistent with those found in previous emotion regulation studies [8], [12], [26]–[29]. Increased thalamus activation in this contrast may reflect a small increase in anticipatory anxiety during reappraisal relative to control, similar to the pattern found for the anxiety vs. control contrast, although subjective anxiety ratings do not support this possibility.

The anxiety vs. regulation contrast revealed significant activation of fear-related brain regions, including the thalamus and amygdala. This result suggests decreased fear-related activity during reappraisal. This finding is similar to that of Herwig et al. [12], who reported decreased amygdala activity in a control vs. no control contrast during expectation of a negative stimulus. The regulation vs. anxiety contrast revealed increased activation of lateral prefrontal regions as well as anterior cingulate cortex (x, y, z = 10, 20, −10). This region of anterior cingulate cortex is often referred to as the ventral medial prefrontal cortex [30], [31]). Many previous studies report have reported that various emotion regulation strategies, such as reappraisal and mindfulness, serve to activate the medial prefrontal, dorsolateral prefrontal, and orbitofrontal cortices [28]. Activation of these regions in the broader context of emotion regulation appears to reflect some degree of common function across various emotion regulation strategies. Increased activation of the ventral medial prefrontal cortex is thought to reflect extinction in the context of fear conditioning. Many studies have reported that increased ventral medial prefrontal cortex activity represses amygdala activity during extinction [32]–[35]. Based on such findings, the ventral medial prefrontal cortex activation we observed during reappraisal may have worked to reduce the amygdala activation associated with anticipatory anxiety. It has been suggested that the ventral part of medial prefrontal cortex is involved with automatic aspects of self-focus [36]. Thus it may cause self-focusing to emotional stimuli, which may be necessary for successful recruitment of reappraisal regulation. By definition, reappraisal is a reinterpretation of emotional stimuli or situation [3], [4] and does not represent a distraction from emotional stimuli or from the situation itself. Someone who intends to regulate emotion by using reappraisal should concentrate their attention on self-relevant incoming emotional stimuli or the situation. The model of cognitive control of emotion suggests that the ventral medial prefrontal cortex might be a mediator in the ventrolateral prefrontal cortex to the amygdala pathways for effective reappraisal [13]. This suggests that it is possible that ventral medial prefrontal cortex activation is not essential for engagement of reappraisal but instead represents a consequence of the successful use of this strategy.

The dorsolateral prefrontal cortex is assumed to play a critical role in the deliberate regulation of emotion. This region is involved in the maintenance of selective attention directed towards a goal during emotion down-regulation [13]. Golkar et al. [9] reported that dorsolateral prefrontal cortex activation is commonly involved in reappraisal of both negative and neutral stimulus content, suggesting that this activity reflects the cognitive processing that is inherent to reappraisal. Activation of the dorsolateral prefrontal cortex might play a similar role across various emotion regulation strategies, as this region has also been implicated in distraction, suppression, and mindfulness [9], [37]–[39]. It therefore seems likely that activation of this region is not unique to reappraisal per se. Because of the relatively sparse connections between the dorsolateral prefrontal cortex and the amygdala, this region is likely to influence the amygdala indirectly [40].

Finally, the present study found increased activation of the orbitofrontal cortex during reappraisal. The orbitofrontal cortex is thought to underlie reward-related decision-making [41], [42] and may also play a role in emotion regulation. Orbitofrontal cortex activity has been associated with the down-regulation of negative emotion [9], [26], [43]. In particular, Golkar et al. [9] suggested that the orbitofrontal cortex is specifically engaged during reappraisal as compared with other emotion regulation strategies, and mediates reappraisal success through subcortical pathways. However, a recent meta-analysis failed to demonstrate that reappraisal consistently recruits the orbitofrontal cortex [28]. The present results include a correlation between successful anxiety reduction via reappraisal and activation of both the anterior cingulate and the orbitofrontal cortices. This correlation supports the hypothesis that successful engagement of reappraisal does involve the orbitofrontal cortex.

In summary, transitory anticipatory anxiety invoked by anticipation of a pain stimulus activates fear circuitry in the brain. In addition, reappraisal strategies to manage such anxiety are associated with activation of medial and lateral prefrontal region activation along with a corresponding suppression of fear circuit activity. Correlational analysis suggested that the orbitofrontal and anterior cingulate cortices are important for successful reappraisal.
